# Isoflurane Increases Neuronal Cell Death Vulnerability by Downregulating miR-214

**DOI:** 10.1371/journal.pone.0055276

**Published:** 2013-02-08

**Authors:** Hailiang Yan, Tao Xu, Hongfeng Zhao, Kuo-Chieh Lee, Hoau-Yan Wang, Yan Zhang

**Affiliations:** 1 State Key Laboratory of Biomembrane and Membrane Biotechnology, College of Life Sciences, Peking University, Beijing, China; 2 Department of Pharmacology, Physiology and Neuroscience, Sophie Davis School of Biomedical Education/City University of New York Medical School, New York, New York, United States of America; 3 Beijing No.4 High School, Beijing, China; 4 Department of Urology, Peking University People’s Hospital, Beijing, China; Massachusetts General Hospital, United States of America

## Abstract

Since accumulating evidence suggests the application of anesthetics may increase the risk of Alzheimer’s disease (AD), we investigated the cytotoxicity of inhaled general anesthesia in neurons and its underlying mechanism. Using primary cultured rat hippocampal neurons as the study model, here we show that isoflurane increases vulnerability to intracellular or extracellular amyloid β with or without serum deprivation. This isoflurane-induced effect is mediated by the downregulation of miR-214 level that lead to an elevated expression of Bax, a prominent target for miR-214. We conclude that isoflurane increases cell death in the presence of amyloid β by increasing Bax level through downregulating miR-214. Our data provide a new insight for inhaled anesthetics toxicity and indicate a possible mechanistic link between anesthetic application and neurodegenration in AD.

## Introduction

Alzheimer’s disease (AD) is a progressive neurodegenerative disease characterized by senile plaques and neurofibrillary tangles in elderly [1]. With increasing number of older people worldwide undergo surgery every year, accumulating evidence indicates the association between anesthetic application and risk for AD. Inhaled anesthetics can impair cognitive function especially in aged individuals [2] and are associated with neuronal damage and prolonged cognitive impairment [2], [3], [4], [5], [6], [7], [8], [9], [10], [11], [12], [13]. While the age of AD onset has been linked to surgery history in which inhaled anesthetics was used [14], [15], [16], [17], there is also evidence indicates that there is no correlation between the usage of general anesthesia and AD [18]. Hence, current clinical data are inconsistent in associating inhaled general anesthesia and risk for AD.

Despite the conflicting clinical evidence on the risk of AD after the usage of inhaled anesthetics, potential mechanisms responsible for neuronal damage following inhaled anesthetia have been suggested. In accord, some anesthetics promote protein misfolding and aggregation, one of the mechanisms associating with cell death in neurodegenerative diseases [12], [19], [20]. Inhaled anesthetics are small haloalkanes or haloethers which can bind to hydrophobic cavities of proteins [21], [22], [23] and have the ability to increase the levels of small oligomers [24] which may include amyloid β (Aβ) peptides aggregates, the main component in senile plaques. Isoflurane was shown to alter Aβ precursor protein (APP) processing, upregulate Aβ production and induce apoptosis in H4 neuroglioma cells [12]. In APP-transfected H4 cells, desflurane and hypoxia can also increase Aβ production and caspase activation [25]. Nuclear magnetic resonance (NMR) data indicate that halothane, isoflurane and desflurane interact with Aβ that leads to its oligomerization [26], [27]. Similarly, inhaled anesthesia increased hyperphosphorylated tau, the primary component of neurofibrillary tangles [28], [29], [30], [31], [32], [33], although there is evidence that the hypothermia, which is induced by anesthesia, rather than anesthetics themselves, causes tau hyperphosphorylation [28]. Further, recent data show that intravenous anesthetic propofol and inhaled anesthetic isoflurane both promote tau hyperphosphorylation [34], [35]. Taken together, our understanding of how anesthetics increase neuronal cell death remains very limited.

In our present study, we examined the underlying mechanism reponsible for isoflurane-induced neuronal cell death when combined with Aβ or serum deprivation insults in cultured primary rat neurons. We found that isoflurane decreased the level of miR-214, which inhibited Bax activation. Therefore, isoflurane enhanced Aβ cytotoxicity through downregulating miR-214.

## Results

### Isoflurane Increased Aβ and Serum Deprivation Cytotoxicity

**Figure 1:**
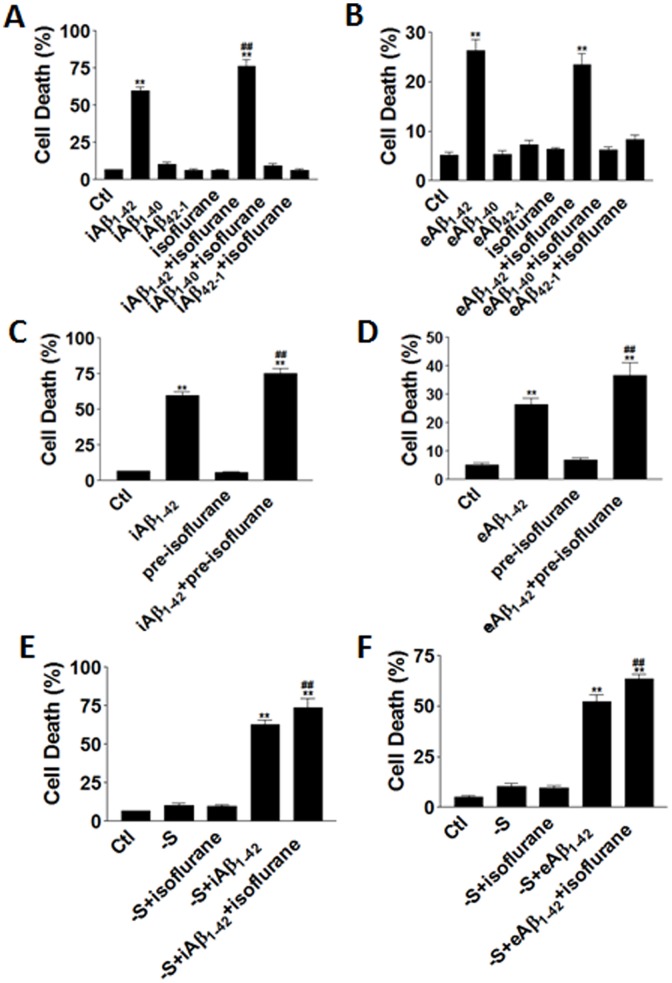
Isoflurane increased Aβ and serum deprivation cytotoxicity. (**A**) Isoflurane enhanced microinjected intracellular Aβ (iAβ)_1-42_, iAβ_1-40_ and iAβ_42-1_ peptide toxicity in primary rat neurons. Cell death levels were measured by TUNEL staining. (**B**) Isoflurane did not enhance extracellular Aβ (eAβ)_1-42_, eAβ_1-40_ and eAβ_42-1_ peptide toxicity in primary rat neurons. (**C**) Pre-treatment of isoflurane for 3 hours increased iAβ_1-42_ peptide toxicity. (**D**) Pre-treatment of isoflurane for 3 hours increased eAβ_1-42_ peptide toxicity. (**E**) Isoflurane enhanced the toxicity of the combination of iAβ_1-42_ peptide and serum deprivation (-S). (**F**) Isoflurane enhanced the toxicity of the combination of eAβ_1-42_ peptide and serum deprivation (-S). Data represent Means+SE (n = 200 for each group). **: p<0.01 compared with the control; ##: p<0.01 compared with the Aβ group.

To further investigate the mechanisms of general anesthesia induced cytotoxicity in cultured primary rat neurons, we treated the cells in a closed chamber with incubation of 0.5% isoflurane for 3 hours. Simultaneously, the neurons were insulted with intracellular Aβ_1-42_ (iAβ), extracellular Aβ_1-42_ (eAβ), or in combination with serum deprivation (-S). In the un-insulted neurons, isoflurane did not induce significant cell death 24 hours after the treatment (Fig. 1A). In contrast, isoflurane markedly increased cell death in neurons that were microinjected with Aβ_1-42_ peptide (iAβ_1-42_) when compared with neurons with iAβ_1-42_ alone (Fig. 1A). However, when neurons were treated with Aβ_1-42_ peptide in culture medium (eAβ_1-42_), isoflurane did not enhance cell death compared with eAβ_1-42_ alone (Fig. 1B). In contrast to iAβ_1-42_ or eAβ_1-42_, iAβ_1-40_ and eAβ_1-40_ or the control reversed peptides iAβ_42-1_ and eAβ_42-1_ did not alter the cell viability (Fig. 1A and 1B). Application of isoflurane did not change the cell death in the presence of iAβ_1-40_, eAβ_1-40_, iAβ_42-1_ and eAβ_42-1_ (Fig. 1A and 1B). When neurons were pre-incubated with isoflurane for 3 hours and then insulted with either iAβ_1-42_ or eAβ_1-42_ peptide, isoflurane pre-treatments significantly increased the cell death (Fig. 1C and 1D). We also examined the cytotoxicity of isoflurane in the presence of serum deprivation. Our results showed that with serum deprivation for 3 days, application of isoflurane for 3 hours did not alter cell death levels (Fig. 1E). Isoflurane enhanced cell death significantly only when iAβ_1-42_ or eAβ_1-42_ peptides were combined with serum deprivation (Fig. 1E and 1F).

### Bax Levels were Enhanced with Isoflurane Treatment

**Figure 2:**
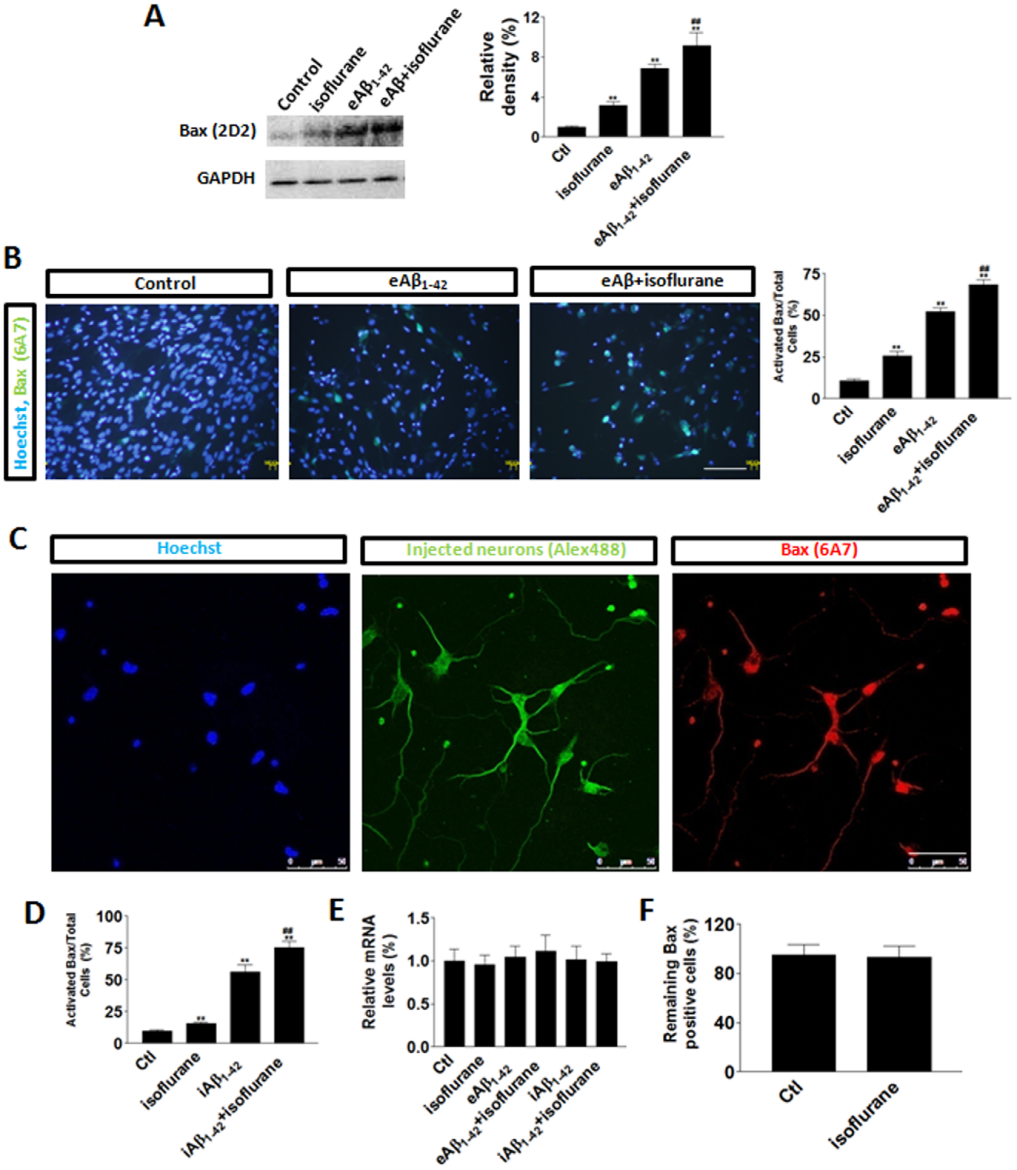
Bax was upregulated by isoflurane treatment. (**A**) Left panel: An example of western blot for total Bax (indicated by 2D2 antibody) suggested that total Bax levels were increased by isoflurane in the absence or presence of eAβ_1-42_ peptide. Right panel: Quantitative data showed that isoflurane significantly enhanced total Bax levels. Data represent Means±SE (n = 3 repeats for each group). (**B**) Left panel: Representative images showed that activated Bax (indicated by 6A7 antibody) was upregulated by isoflurane. Scale bar: 100 µm. Right panel: Quantitative data showed that isoflurane significantly enhanced total bax levels. (**C**) Representative images showed microinjected neurons (indicated by injection marker dye Alex488) with iAβ_1-42_ peptide and activated Bax (6A7) staining. Scale bar: 50 µm. (**D**) Quantitative data showed that isoflurane significantly enhanced total bax levels. (**E**) Quantitative RT-PCR results showed that there was no significant difference between isoflurane treatment groups on Bax mRNA. (**F**) Quantitative data showed that isoflurane did not alter exogenous Bax levels at 12 hours after the treatment. Data represent Means±SE (n = 200 cells for each group). **: p<0.01 compared with the control. ##: p<0.01 compared with the Aβ group.

Pre-incubation of isoflurane potentiated the toxicity induced by both iAβ_1-42_ or eAβ_1-42_ peptides in neurons (Fig. 1C and 1D). To investigate the underlying mechanism responsible for the elevated cytotoxicity of isoflurane treatment, we examined the protein levels of Bax in the absence or presence of eAβ_1-42_ peptide application. Our Western blot data show that isoflurane markedly increased total Bax levels in the absence and presence of eAβ_1-42_ peptide (Fig. 2A). Using an antibody specifically detects the activated Bax (6A7), our results showed that isoflurane also increased activated Bax in the absence and presence of eAβ_1-42_ (Fig. 2B). When neurons were microinjected iAβ_1-42_ peptide (Fig. 2C), the level of activated Bax was increased as indicated by increased 6A7 staining in more neurons (Fig. 2D). The increased activated Bax level is not mediated by the elevated Bax mRNA since quantitative RT-PCR data indicated that there was no significant difference in Bax mRNA levels in these treatment groups (Fig. 2E). These data suggest that Bax protein levels might be modified by anesthetics at post-transcriptional levels. To rule out the possibility that isoflurane might decrease Bax degradation to enhance Bax level, we microinjected Bax-EGFP fusion protein expressing construct to primary neurons. A successful injection was indicated by the presence of Dextran Texas Red (DTR) staining. The cells were then treated with isoflurane for 3 hours immediately after Bax-EGFP injection. At 12 hours after anesthetics treatment, the numbers of EGFP positive cells versus DTR positive cells were counted. Our results showed that there was no significant difference between the treatment groups compared with the control group (Fig. 2F), suggesting that isoflurane did not affect Bax degradation.

### Isoflurane Increased Bax Level through Downregulating miR-214

**Figure 3:**
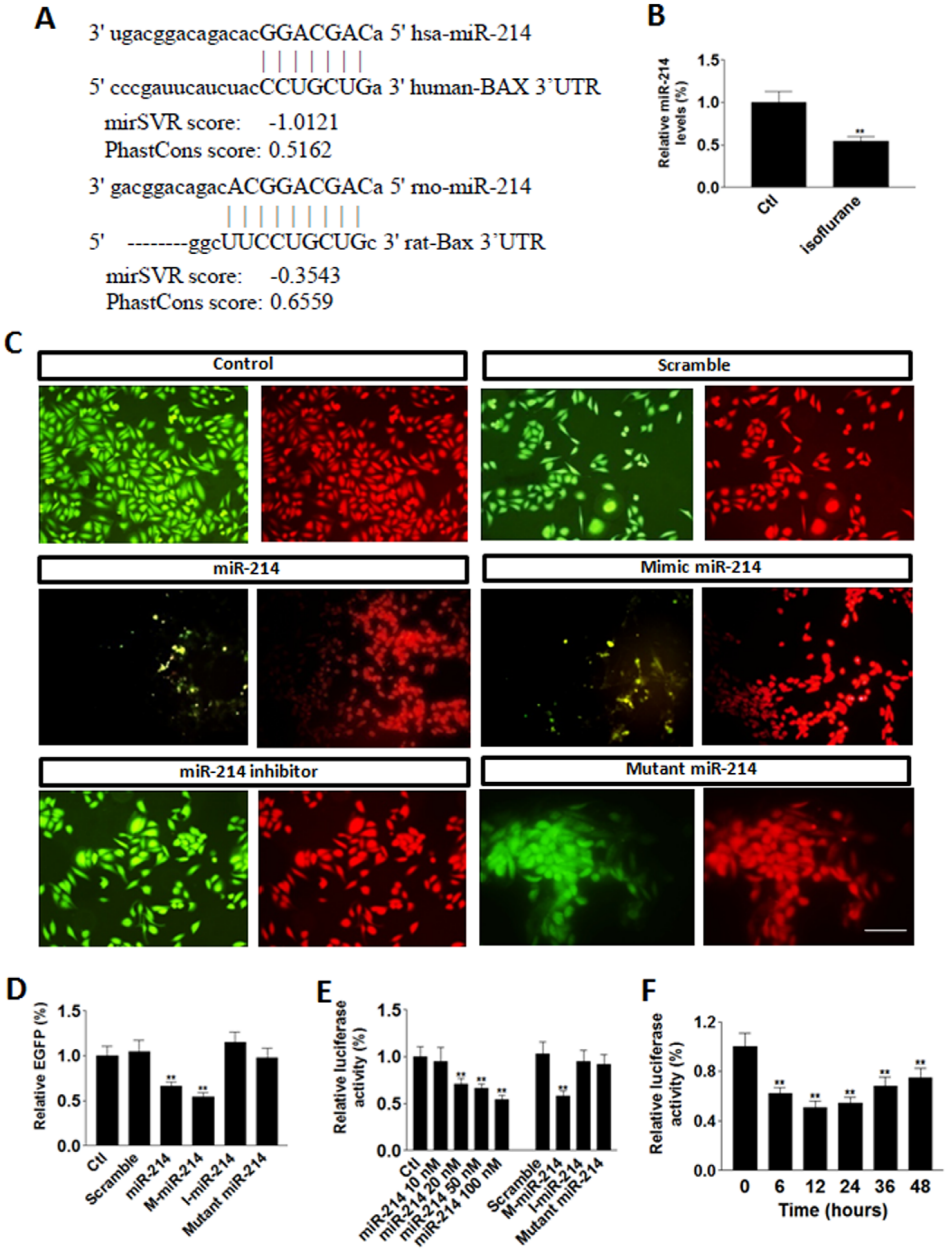
Isoflurane decreased miR-214. (**A**) Sequence alignment of human and rat miR-214 and Bax 3′UTR. (**B**) Levels of relative miR-214 in isoflurane-treated primary rat neurons. (**C**) Representative images showed that in SH-Sy5y cells, the levels of reporter Bax 3′UTR regulated EGFP (green fluorescent) were regulated by miR-214 and its variants. RFP (red fluorescent) indicated the transfected cells. Scale bar: 50 µm. (**D**) Quantitative data showed that miR-214 decreased Bax expression. M-miR-214: mimic miR-214; I-miR-214: miR-214 inhibitor. (**E**) miR-214 decreased Bax expression in a dose-dependent manner. (**F**) The time course of miR-214 in suppression of Bax expression. Data represent Means±SE (n = 200 cells for each group). **: p<0.01 compared with the control.

To further investigate the mechanism responsible for isoflurane-induced increase in Bax level, we performed microRNA array assay in the neuronal extracts from control and isoflurane-treated primary neurons. Among the microRNAs altered by isoflurane application, miR-214 was significantly downregulated (Fig. 3B). In accord with the notion that miR-214 level may be releated to Bax abundance, Bax 3′UTR was predicted to be a target of miR-214 (Fig. 3A). To this end, we transfected the neuroblastoma SH-Sy5y cells with human Bax 3′UTR regulated EGFP construct and co-transfected with control, scramble microRNA, miR-214, mimic miR-214, miR-214 inhibitor or mutant miR-214, respectively. The transfection efficiency was indicated by RFP (Fig. 3C). Our data showed that the relative number of EGFP positive cells in RFP positive cells was decreased in the miR-214 and mimic miR-214 transfected groups, but not in the scramble, miR-214 inhibitor or mutant miR-214 groups (Fig. 3D), suggesting that miR-214 downregulated Bax expression. Similarly, Bax 3′UTR regulated luciferase assay showed miR-214 from 20 to 100 nM efficiently decreased Bax expression (Fig. 3E). Time course of miR-214 indicated that maximal inhibition happened at 12–24 hours after transfection, although the effect might last for at least 48 hours (Fig. 3F).

**Figure 4:**
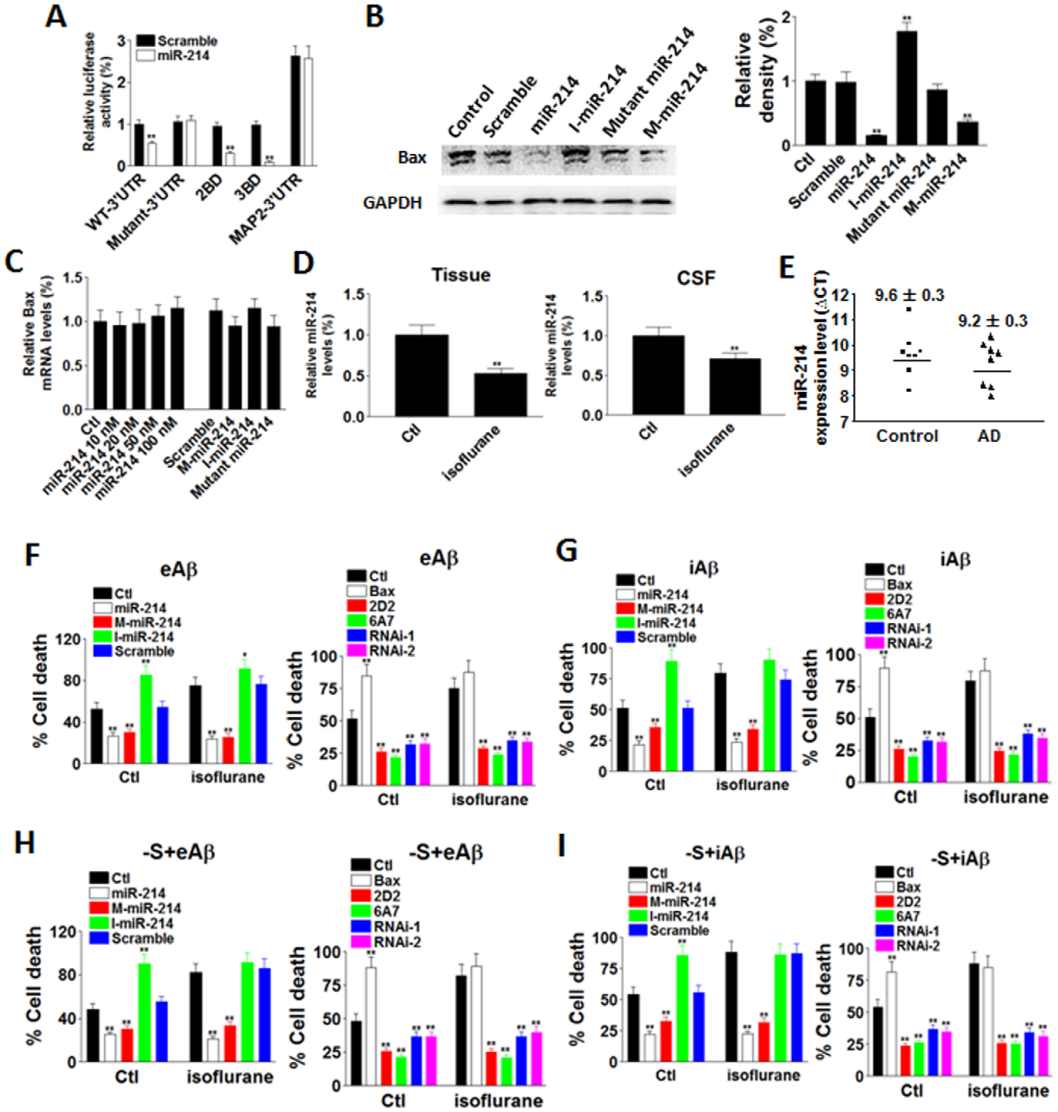
Isoflurane regulated cell death through miR-214. (**A**) miR-214 specifically acted on Bax 3′UTR. (**B**) In SH-Sy5y cells, miR-214 inhibited total Bax (indicated by 2D2 antibody) expression. (**C**) miR-214 did not alter Bax mRNA levels. (**D**) In the rats treated with inhaled isoflurane, miR-214 levels were decreased in the brain tissues and CSF. (**E**) The expression level of miR-214 in matched Alzheimer’s disease and control pairs. RT-PCR results for hippocampal tissues from 8 matched control-AD pairs with miR-214 showed that there was no discernible difference detected between two diagnostic groups (p = 0.38281, two-tailed Student’s t-test). (**F**) Left panel: in cultured primary rat neurons, microinjected miR-214 or mimic miR-214 (M-miR-214) reversed cell death induced by isoflurane in the presence of eAβ_1-42_. Right panel: Bax neutralizing antibodies (2D2 and 6A7) and Bax siRNAs (RNAi-1, RNAi-2) blocked cell death induced by isoflurane in the presence of eAβ_1-42_. (**G**) Left panel: microinjected miR-214 or mimic miR-214 (M-miR-214) reversed cell death induced by isoflurane in the presence of iAβ_1-42_. Right panel: Bax neutralizing antibodies (2D2 and 6A7) and Bax siRNAs (RNAi-1, RNAi-2) blocked cell death induced by isoflurane in the presence of iAβ_1-42_. (**H**) Left panel: microinjected miR-214 or mimic miR-214 (M-miR-214) reversed cell death induced by isoflurane in the presence of eAβ_1-42_ and serum deprivation (-S). Right panel: Bax neutralizing antibodies (2D2 and 6A7) and Bax siRNAs (RNAi-1, RNAi-2) blocked cell death induced by isoflurane in the presence of eAβ_1-42_ and serum deprivation (-S). (**I**) Left panel: microinjected miR-214 or mimic miR-214 (M-miR-214) reversed cell death induced by isoflurane in the presence of iAβ_1-42_ and serum deprivation (-S). Right panel: Bax neutralizing antibodies (2D2 and 6A7) and Bax siRNAs (RNAi-1, RNAi-2) blocked cell death induced by isoflurane in the presence of iAβ_1-42_ and serum deprivation (-S). Data represent Means±SE (n = 200 cells for each group). **: p<0.01 compared with the control.

To further confirm the role of miR-214 in regulating Bax level, mutant Bax 3′UTR was made by altering 2 nucleotides at WT Bax 3′UTR region. When mutant Bax 3′UTR was co-transfected into SH-Sy5y cells with either scramble or miR-214 sequence, luciferase reportor assay showed no significant difference between scramble and miR-214 transfected groups (Fig. 4A), suggesting that miR-214 targeted Bax 3′UTR specifically. To further proof that miR-214 regulate Bax 3′UTR selectively, microtubule-associated protein 2 (MAP2) 3′UTR was co-transfected with scramble or miR-214 sequence. Our data prove no noticeable different between scramble and miR-214 groups (Fig. 4A). Since microRNAs bind to mRNAs at specific regions (binding domain, BD), we made Bax 3′UTR with double BD (2BD) and triple BD (3BD). Our data showed that miR-214 inhibits Bax expression increased propotionally with 2BD or 3BD construct (Fig. 4A), thus confirming that miR-214 targeted Bax and downregulated its expression. Western blot data showed that in SH-Sy5y cells, miR-214 and mimic miR-214 decreased Bax level (Fig. 4B), while Bax mRNA levels were not altered by miR-214 (Fig. 4C). These data together indicate that miR-214 regulated Bax mRNA translation. The relevance of isoflurane *in vivo* was tested in the rats treated with inhaled 1% isoflurane for 3 hours and the levels of miR-214 were then measured in the whole brain tissues and CSF. Our data indicated that following anesthetic treatment, miR-214 level was decreased significantly in both brain tissues and CSF (Fig. 4D). To assess whether AD affects the level of miR-214, we measured miR-214 expression in the postmortem tissues from AD patients or age matched controls. The RT-PCR results of hippocampal tissues from 8 matched control-AD pairs with miR-214 showed that there was no discernible difference detected between two diagnostic groups (p = 0.38281, two-tailed Student’s t-test). There are no significant correlation between miR-214 expression and age (R^2^ = −0.69457) or postmortem intervals (R^2^ = −0.39699) (Fig. 4E).

We then verified the effect of miR-214 on cell death in rat primary neurons. Neurons were microinjected with miR-214, mimic miR-214, miR-214 inhibitor or scramble sequence and insulted with eAβ_1-42_. The cells were treated with isoflurane for 3 hours and after 24 hours of treatment, the percentages of cell death were measured. Our data showed that cell death was significantly inhibited by miR-214 or mimic miR-214 injection, and enhanced by miR-214 inhibitor injection (Fig. 4F). In parallel, we microinjected Bax construct, Bax neutralizing antibodies 2D2 and 6A7 [36] and siRNAs to Bax. Our results indicated that while Bax increased cell death significantly, its neutralizing antibodies and siRNAs decreased cell death (Fig. 4F). Our data indicate that miR-214 can reverse isoflurane-induced cell death in the presence of eAβ_1-42_, which can be mimicked by Bax suppression. Similarly, the cell death induced by isoflurane with iAβ_1-42_ and the combination of serum deprivation and Aβ can also be reversed by miR-214 (Fig. 4G–I), suggesting that miR-214 is indeed involved in isoflurane cytotoxicity.

## Discussion

The mechanisms leading to neurodegeneration in the neurodegenerative disease such as AD are of intense interest, among which amyloid-β is generally considered to be a pathological marker for AD. In accord with the notion that amyloid-β promotes neurodegeneration, our previous studies demonstrate that iAβ_1-42_ and eAβ_1-42_ can induced neuronal cell death through activating Bax [36], [37]. In the present study, we show that the commonly used inhaled anesthetic, isoflurane, although do not enhance cell death by themselves, increases vulnerability of the pre-exposed neurons to both iAβ_1-42_ and eAβ_1-42_ insults. Additionally, our data indicate that isoflurane increases both total Bax and activated Bax levels. These data together suggest that inhaled anesthetics do not directly alter cell viability but promote cell vulnerability to Aβ insult. Since isoflurane induces Bax activation without showing significant cell death, our data suggest that Bax activation by the inhaled anesthetics may have de-stabilized mitochondria which in turn promote cell vulnerability to amyloid-β insult and the eventual cell death. Interestingly, isoflurane does not increase neuronal vulnerability to serum deprivation alone although the underlying mechanism for this differential effect remains obscured. Similar to our results, earlier studies also support inhaled anesthetics may promote apoptosis. In postnatal day 7 rat pups, combined application of isoflurane, midazolam and nitrous oxide for 6 hours induces excessive neuronal apoptosis throughout the brain such as the hippocampus and cerebral cortex [7]. In cultured cells, isoflurane, isoflurane with nitrous oxide, sevoflurane, desflurane with hypoxia all have shown to induce apoptosis and increase Aβ formation [25], [38], [39], [40], [41].

**Figure 5:**
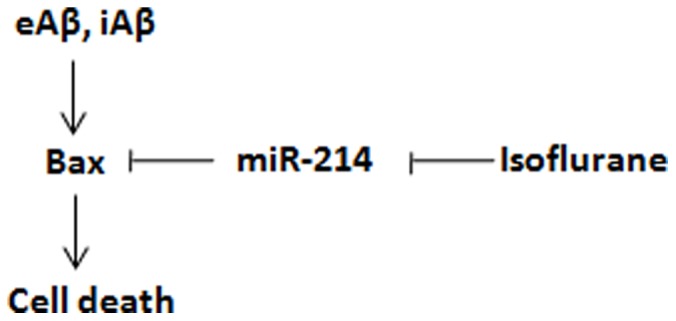
Schematic drawing for isoflurane cytotoxicity mechanism.

It has been proposed that general anesthesia induces apoptosis by suppressing neuronal activity, that in turn inhibits neurotrophic factor secretion locally [42], [43]. Caspase involvement in anesthesia-induced apoptosis has also been implicated [25]. Although an earlier report indicate that 2% isoflurane increases Bax mRNA levels [44], we did not observe any discernible changes in Bax mRNA following incubation with 0.5% isoflurane. Further investigation of potential underlying mechanism reveals that isoflurane decreases miR-214 level. miR-214 targets Bax 3′UTR and downregulates Bax expression by inhibiting its mRNA translation. Therefore, isoflurane induces cytotoxicity and neuronal cell death in the presence of Aβ through downregulating miR-214 (Fig. 5). This is the first time, to our knowledge, that inhaled anesthetics induces cell death by regulating microRNAs is reported. In addition to Bax expression that may be regulated by miR-214, one single nucleotide polymophism (SNP) in presenilin-1 (PS1) (rs63750082) was shown to create a potential target site for miR-214 [45]. This may result in the downregulation of PS1 by miR-214 and thereby reduces the production of Aβ and lessens AD pathology. However, our results in the postmortem hippocampal tissues from 8 matched control-AD pairs indicate that there is no discernible change in the miR-214 level in AD. Given that a higher Aβ pathology in the AD brain, our results predict that inhaled anesthetics would have hastened neurodegeneration in AD subjects.

There is evidence that desflurane and isoflurane interact with both nicotinic and muscarinic acetylcholine receptors (AchR) in a dose-dependent manner [46]. Isoflurane treatment significantly decreases the level of choline acetylase (ChAT), but not α7 nicotinic AchR in mouse brains [47]. Chronic application of isoflurane also alters the level of muscarinic AchR in the hippocampus [48]. Therefore, it is reasonable to hypothesize that symptoms related to postoperative cognitive dysfunction are possible consequence of inhaled anesthetics interacting with AchR and suppressing central cholinergic transmission [49]. Furthermore, the role for the defected cholinergic function in AD pathogenesis is well-supported by many studies [50], it is also possible that inhaled anesthetics increase AD incidence through inhibiting cholinergic function. Together with our data indicating that isoflurane reduces miR-214 to increase cell death by promoting Aβ_1-42_ cytotoxicity, there data support the idea that inhaled anesthetics may increase the risk of AD [33]. Most importantly, our finding in this study suggests that therapies that increase miR-214 may be used to reduce the neurotoxicity and post-operative cognitive dsyfunction following the usage of inhaled anesthetics, especially in AD patients.

## Materials and Methods

### Cell Culture

Primary neurons were cultured from new born Sprague Dawley (SD) rat hippocampus. The protocol has been approved by the regulations of Peking University Institutional Animal Care and Use Committee (IACUC). In brief, the fresh hippocampal tissues were dissociated with 0.25% trypsin (Invitrogen, Carlsbad, CA), which was then inactivated by 10% decomplemented fetal bovine serum (FBS, HyClone, Logan, UT). The mixture was triturated through pipette to make a homogenous mixture. After filtering the mixture through 70 µm sterilized filters, the flow-through was centrifuged. The pellet was then washed once by phosphate buffered saline (PBS, 0.14 M NaCl, 0.003 M KCl, 0.01 M Na_2_HPO_4_, 0.002 M KH_2_PO_4_, pH7.2) and once by DMEM in Earle’s balanced salt solution containing 0.225% sodium bicarbonate, 1 mM sodium pyruvate, 2 mM _L_-glutamine, 0.1% dextrose, 1x antibiotic Pen-Strep (all from Invitrogen, Carlsbad, CA) with 5% FBS. Cells were then plated on poly-_L_-lysine (Sigma, St. Louis, MO) coated plates or glass coverslips at the density of 5×10^4^ cells/ml. Neurons were incubated at 37°C in DMEM without phenol red with 5% FBS and with 5% circulating CO_2_. Cytarabine was added to culture media 24 hours after plating at 10 µM to inhibit dividing cell growth. Medium was changed every 48 hours. SH-Sy5y cells were maitained in DMEM/F12 with 10% FBS.

### Cell and Animal Treatments

Aβ peptides (Bachem, King of Prussia, PA) were dissolved in sterile distilled water at 25 µM and immediately frozen at -20°C. IAβ peptides were delivered into neurons by microinjection at 10 nM at the cytosolic area. EAβ peptides were added to the culture medium at 1 µM. For anesthetics treatment on cultured cells, 0.5% isoflurane (l-chloro-2,2,2-trifluoroethyl difluoromethyl ether) (Guoyao Group, Beijing, China) in humidified air with 5% circulating CO_2_ was applied to a closed incubator 37°C for 3 hours. For anesthetics treatment on animals, SD rats (male, around 250 g) were anesthetized with humidified 1% isoflurane in 30% O_2_ balanced by N_2_ for 3 hours in a closed chamber. After exposure, the animals were returned to their cages. For experiments described in Fig. 3, human miR-214, scramble microRNA control, mimic miR-214, miR-214 inhibitor and mutant miR-214 (5′-ACAG**AC**GGCACAGACAGGCAGU-3′), and for experiments described in Fig. 4F–I, rat miR-214, scramble microRNA control, mimic miR-214, miR-214 inhibitor were all purchased from Qiagen (Hilden, Germany). For experiments described in Fig. 4A, several altered human Bax 3′UTR sequences used were as following: mutant Bax 3′UTR: 5′-CCCGAUUCAUCUACCC**GAU**UGA-3′; 2BD: 5′-CCCGAUUCAUCUAC**CCUGCUGCCUGCUG**A-3′; 3BD: 5′-CCCGAUUCAUCUAC**CCUGCUGCCUGCUGCCUGCUG**A-3′; MAP2-3′UTR: 5′-CATATTCATTCTTTACAAACCATAG-3′. All WT and modified 3′UTR sequences were cloned into pLenti-Luc-UTR vector backbone (Abm, Richmond, BC, Canada). MicroRNAs were transfacted into SH-Sy5y cells with Lipofectamine 2000 (Invitrogen, Carlsbad, CA) and microinjected into primary rat neurons. MicroRNA levels were measured by CapitalBio (Beijing, China) microarray service with 3 repeats by using GeneChip microRNA 2.0 (Affymetrix). miR-214 in the brain tissue and CSF was measured by TaqMan MicroRNA assay kit for rno-miR-214 according to the manufacturer’s instruction (Life Technologies Corp. Shanghai, China). All Bax siRNAs (Qiagen, Hilden, Germany) were diluted into 5 nM before injection described by the manufacturer. The silencing efficiency and off-target effects of all siRNAs were verified by Qiagen.

### Microinjection

Thin-walled Borosilicate glass capillaries (outer diameter = 1.0 mm, inner diameter = 0.5 mm) with microfilament (MTW100F-4, World Precision Instrument, Sarasota, FL) were pulled with a Flaming/Brown Micropipette Puller (P-97, Sutter, Novato, CA) to obtain injection needles with a tip diameter of ∼0.5 µm. Microinjections were performed in the cytosol of each cell using the Eppendorf Microinjector FemtoJet and Eppendorf Micromanipulator (Eppendorf, Hamburg, Germany). Neurons were injected with 25 fl/shot at an injection pressure of 100 hPa, a compensation pressure of 50 hPa, and an injection time of 0.1 seconds, at the soma areas. The solutions were injected at the indicated concentrations with 100 µg/ml dextran Texas Red (DTR, MW: 3000, Molecular Probes, Eugene, OR) or Alex488 (Molecular Probes, Eugene, OR) as fluorescent markers to recognize the injected cells. Approximately 90% neurons survive the injections for at least 16 days [51].

### Measurement of Neuronal Cell Death

Cells were fixed in fresh 4% paraformaldehyde, 4% sucrose in PBS for 20 minutes at room temperature and permeablized in 0.1% Triton X-100, 0.1% sodium citrate in PBS for 2 minutes on ice. Terminal deoxynucleotidyl transferase-biotin dUTP nick-end labeling (TUNEL) staining was performed using the *in situ* cell death detection kit I as described by the manufacturer (Roche, Quebec, Canada). The coverslips were then washed once in distilled water for 5 minutes and mounted on glass slides to be observed under a fluorescence microscope. For the non-injected cells, the percentage of cell death was determined by the ratio of the number of TUNEL-positive cells over the total of 100 cells in one count. The average of 5 counts was calculated as the percentage of neuronal cell death in a certain treatment. For the injected cells, the percentage of cell death was determined as the ratio of the number of DTR-TUNEL-double-positive cells over the total number of DTR-positive cells.

### Immunostaining

Cells were permeablized in PBS-Triton at 4°C, blocked 10% donkey serum at room temperature, followed by incubation with anti-activated Bax antibody (6A7, R&D, Minneapolis, MN, 1∶200) at 4°C for 24 hours. Cy2 or Cy3-conjugated donkey anti-rabbit antibody was applied as the secondary antibody. The nuclei were then staining by Hoechst 33258 (1 µg/ml, Sigma, St. Louis, MI) for 15 minutes in dark. The coverslips were mounted with Immunon™ mounting medium (Shandon, Pittsburgh, PA) onto glass slides the results were analyzed by using fluorescence microscope (Olympus BH2-RFCA, Olympus, Tokyo, Japan) with digital camera (Olympus DP70 Digital Microscope Camera, Olympus, Tokyo, Japan).

### Postmortem Tissues

This study protocol conformed to the Helsinki (the 4^th^ amendment) as reflected in a prior approval by the institution’s human research committee. Postmortem brain tissues (frontal cortex) from patients with clinically diagnosed sporadic AD (n = 8, age range = 56–91, mean: 71.1±4.7) and control tissues from normal, age-matched, neurologically normal (n = 8, age range = 55–99, mean: 72.0±5.7) individuals were obtained from the University of Pennsylvania Brain Tissue Resource Center. The postmortem time intervals for collecting these brains were ≤ 15 hr (mean postmortem intervals for collection of AD and control brain samples were 9.6±1.5 hr and 9.6±1.8 hr, respectively). There were no discernible differences between diagnostic groupa in age (p = 0.90141) and postmortem interval (p = 0.98219).

All cases of AD dementia studied met clinical criteria for that disorder specified by NINCDS-ADRDA [52] as determined in consensus conferences after review of medical records, direct clinical assessments, and interviews of care providers. Clinical diagnosis requires that an individual showed clear cognitive decline from his or her previous levels as verified in tests of memory and in least one other cognitive domain (e.g., perceptual speed). The diagnoses were confirmed by postmortem examination of neuritic plaque densities in midfrontal gyrus (dorsolateral prefrontal cortex), superior+inferior temporal gyrus, inferior parietal gyrus, hippocampus, and substantia nigra as specified by the Consortium to Establish a Registry for AD (Mirra et al., 1991). The final diagnoses were consistent with Braak scores for neurofibrillary tangle pathology as recommended by the NIA-Reagan Institute consensus on diagnosis of AD [53]. All postmortem tissues were identified by an anonymous identification number, and experiments were performed as a best matched pair without knowledge of clinical information.

### Tissue Samples and Extraction

Frozen hippocampal formation was lysed in QIAzol lysis reagent (Qiagen, Hilden, Germany) and homogenized with Kontes pestle. Extraction of RNA was performed by using miRNeasy kit (cat# 217004, Qiagen, Hilden, Germany). Briefly, for 700 l of QIAzol, 140 l of chloroform was added and mixed for 15 seconds. After 3 minutes incubation at room temperature, the samples were centrifuged at 12,000 g (4°C) for 15 min. The upper aqueous layer containing RNA was transferred to another microcentrifuge tube for RNA extraction. Total RNA was solubilized in nuclease free water (cat# AM9932, Ambion) purified using RNeasy Mini columns (Qiagen, Hilden, Germany) according to manufacturer instructions. Nucleic acid concentrations were determined by Qubit fluorometer (cat# Q32857, Invitrogen, Carlsbad, CA) and samples were stored at 80°C.

### Real Time PCR (RT-PCR)

For brain tissues, RT-PCR was performed using the QIAgen MicroRNA Reverse Transcription kit (cat# 218161, Miscript II RT). cDNA concentrations were determined by Qubit fluorometer. QIAgen Miscript SYBR Green PCR kit (cat # 218073) was used to detect miR-214 expression. Forward primer Micro RNA 214, 5′-acagcaggcacagacaggcagu-3′, was purchased from QIAgen (cat# MS00031605). The QIAgen small nucleolus RNA primer (RNU 6 cat# MS00031605) was used to detect reference gene. All real time PCR reactions were performed on iCycler Real Time PCR System (BioRad, Hercules, CA) according to manufacturer’s recommendation. MicroRNA quantity in each individual sample was calculated by the delta Ct method.

For RT-PCR with cellular extracts, cells were harvested and total RNA was isolated with TRIGene reagent (GenStar BioSolutions Co., Ltd., Beijing, China). Total RNA (2 µg) were reversely transcribed using TransScript II First-Strand cDNA Synthesis SuperMix (Beijing TransGen Biotech Co., Ltd., Beijing, China). Real-time PCRs were done by using TransStart Green q PCR SuperMix UDG™ (Beijing TransGen Biotech Co., Ltd., Beijing, China). Sequences of primers for Bax used were as following: forward: (5′-GCAGAGGATTGCTGATG-3′); and reverse: (5′-CTCAGCCCATATTCTTCCAG-3′). Real-time PCR quantifications were run in triplicate for each sample and the average were determined. In order to use the comparative Ct method for relative quantification, the amplification efficiency of target and housekeeping gene must be approximately equal. Quantification was done using the comparative Ct method, expression levels for the target gene was normalized to the GAPDH of each sample [2^−△Ct^ = 2^−(Ct(target gene)−Ct(GAPDH))^]. Amplification was done for 45 cycles at 95°C for 30 s, 59°C for 30 s, 72°C for 30 s, 95°C for 1 min, 59°C for 30 s and 95°C for 30 s.

### Western Blots

Neuronal proteins were extracted in the cell lysis buffer (50 mM Tris, pH8.0, 150 mM NaCl, 1% NP-40, 0.1% SDS) and protein concentrations were measured by bicinchoninic acid (BCA) assay (Pierce, Rockford, IL). Protein extracts were denatured at 100°C for 5 minutes and separated on 15% sodium dodecyl sulphate-polyacrylamide gel electrophoresis (SDS-PAGE) at 70 volts for about 2 hour. Proteins were transferred to Immobilon-P™ polyvynilidene fluoride (PVDF) membrane (Millipore, Billerica, MA) at 100 milliamps for 2 hours. The membrane was blocked with 5% non-fat milk in Tris buffered saline (TBS) with 0.1% tween20 (TBS-T) at room temperature for 1 h. Anti-Bax (2D2, R&D, Minneapolis, MN) and GAPDH (Sigma, St. Louis, MO) antibodies were diluted at 1∶1000 for Western blots as primary antibodies. After 3 washes of 10 minutes each with TBS-T, goat anti-mouse or anti-rabbit IgG conjugated with horseradish peroxidase (HRP) was added in a dilution of 1∶2500 as the secondary antibody. The secondary HRP was detected by enhanced chemiluminescence. Optical density was analyzed by BioRad ChemiDox (BioRad, Hercules, CA). The relative density was calculated by the total absolute density of Bax/GAPDH.

### Statistical Evaluation

Statistical significance was assessed by one-way analysis of variances (ANOVA). The Sheffé’s test was applied as a *post hoc* for the significant difference shown by ANOVAs. A p value of less than 0.05 was used as an indicative of statistical significance.

## References

[1] Price DL, Sisodia SS, Gandy SE (1995) Amyloid beta amyloidosis in Alzheimer’s disease. Curr Opin Neurol 8: 268–274. 7582041 10.1097/00019052-199508000-00004

[2] Bianchi SL, Tran T, Liu C, Lin S, Li Y, et al (2008) Brain and behavior changes in 12-month-old Tg2576 and nontransgenic mice exposed to anesthetics. Neurobiol Aging 29: 1002–1010. 17346857 10.1016/j.neurobiolaging.2007.02.009 PMC4899817

[3] Muravchick S, Smith DS (1995) Parkinsonian symptoms during emergence from general anesthesia. Anesthesiology 82: 305–307. 7832317 10.1097/00000542-199501000-00039

[4] Culley DJ, Baxter M, Yukhananov R, Crosby G (2003) The memory effects of general anesthesia persist for weeks in young and aged rats. Anesth Analg 96: 1004–1009, table of contents. 10.1213/01.ANE.0000052712.67573.12 12651650

[5] Culley DJ, Baxter MG, Crosby CA, Yukhananov R, Crosby G (2004) Impaired acquisition of spatial memory 2 weeks after isoflurane and isoflurane-nitrous oxide anesthesia in aged rats. Anesth Analg 99: 1393–1397; table of contents. 10.1213/01.ANE.0000135408.14319.CC 15502036

[6] Culley DJ, Baxter MG, Yukhananov R, Crosby G (2004) Long-term impairment of acquisition of a spatial memory task following isoflurane-nitrous oxide anesthesia in rats. Anesthesiology 100: 309–314. 14739805 10.1097/00000542-200402000-00020

[7] Jevtovic-Todorovic V, Hartman RE, Izumi Y, Benshoff ND, Dikranian K, et al (2003) Early exposure to common anesthetic agents causes widespread neurodegeneration in the developing rat brain and persistent learning deficits. J Neurosci 23: 876–882. 12574416 10.1523/JNEUROSCI.23-03-00876.2003 PMC6741934

[8] Kvolik S, Glavas-Obrovac L, Bares V, Karner I (2005) Effects of inhalation anesthetics halothane, sevoflurane, and isoflurane on human cell lines. Life Sci 77: 2369–2383. 15993426 10.1016/j.lfs.2004.12.052

[9] Loop T, Dovi-Akue D, Frick M, Roesslein M, Egger L, et al (2005) Volatile anesthetics induce caspase-dependent, mitochondria-mediated apoptosis in human T lymphocytes in vitro. Anesthesiology 102: 1147–1157. 15915027 10.1097/00000542-200506000-00014

[10] Wei H, Kang B, Wei W, Liang G, Meng QC, et al (2005) Isoflurane and sevoflurane affect cell survival and BCL-2/BAX ratio differently. Brain Res 1037: 139–147. 15777762 10.1016/j.brainres.2005.01.009

[11] Yon JH, Daniel-Johnson J, Carter LB, Jevtovic-Todorovic V (2005) Anesthesia induces neuronal cell death in the developing rat brain via the intrinsic and extrinsic apoptotic pathways. Neuroscience 135: 815–827. 16154281 10.1016/j.neuroscience.2005.03.064

[12] Xie Z, Dong Y, Maeda U, Alfille P, Culley DJ, et al (2006) The common inhalation anesthetic isoflurane induces apoptosis and increases amyloid beta protein levels. Anesthesiology 104: 988–994. 16645451 10.1097/00000542-200605000-00015

[13] Xie Z, Tanzi RE (2006) Alzheimer’s disease and post-operative cognitive dysfunction. Exp Gerontol 41: 346–359. 16564662 10.1016/j.exger.2006.01.014

[14] Breteler MM, van Duijn CM, Chandra V, Fratiglioni L, Graves AB, et al (1991) Medical history and the risk of Alzheimer’s disease: a collaborative re-analysis of case-control studies. EURODEM Risk Factors Research Group. Int J Epidemiol 20 Suppl 2S36–42. 1833352 10.1093/ije/20.supplement_2.s36

[15] Bohnen NI, Warner MA, Kokmen E, Beard CM, Kurland LT (1994) Alzheimer’s disease and cumulative exposure to anesthesia: a case-control study. J Am Geriatr Soc 42: 198–201. 8126336 10.1111/j.1532-5415.1994.tb04952.x

[16] Gasparini M, Vanacore N, Schiaffini C, Brusa L, Panella M, et al (2002) A case-control study on Alzheimer’s disease and exposure to anesthesia. Neurol Sci 23: 11–14. 12111615 10.1007/s100720200017

[17] Lee TA, Wolozin B, Weiss KB, Bednar MM (2005) Assessment of the emergence of Alzheimer’s disease following coronary artery bypass graft surgery or percutaneous transluminal coronary angioplasty. J Alzheimers Dis 7: 319–324. 16131734 10.3233/jad-2005-7408

[18] Zuo C, Zuo Z (2010) Spine Surgery under general anesthesia may not increase the risk of Alzheimer’s disease. Dement Geriatr Cogn Disord 29: 233–239. 20375503 10.1159/000295114 PMC2865396

[19] Eckenhoff RG, Johansson JS, Wei H, Carnini A, Kang B, et al (2004) Inhaled anesthetic enhancement of amyloid-beta oligomerization and cytotoxicity. Anesthesiology 101: 703–709. 15329595 10.1097/00000542-200409000-00019

[20] Ghirlanda G, Hilcove SA, Pidikiti R, Johansson JS, Lear JD, et al (2004) Volatile anesthetic modulation of oligomerization equilibria in a hexameric model peptide. FEBS Lett 578: 140–144. 15581631 10.1016/j.febslet.2004.10.087

[21] Eckenhoff RG, Johansson JS (1997) Molecular interactions between inhaled anesthetics and proteins. Pharmacol Rev 49: 343–367. 9443162

[22] Eckenhoff RG (2001) Promiscuous ligands and attractive cavities: how do the inhaled anesthetics work? Mol Interv 1: 258–268. 14993365

[23] Liu R, Loll PJ, Eckenhoff RG (2005) Structural basis for high-affinity volatile anesthetic binding in a natural 4-helix bundle protein. FASEB J 19: 567–576. 15791007 10.1096/fj.04-3171com

[24] Carnini A, Lear JD, Eckenhoff RG (2007) Inhaled anesthetic modulation of amyloid beta(1–40) assembly and growth. Curr Alzheimer Res 4: 233–241. 17627480 10.2174/156720507781077278

[25] Zhang B, Dong Y, Zhang G, Moir RD, Xia W, et al (2008) The inhalation anesthetic desflurane induces caspase activation and increases amyloid beta-protein levels under hypoxic conditions. J Biol Chem 283: 11866–11875. 18326038 10.1074/jbc.M800199200 PMC2335348

[26] Mandal PK, Pettegrew JW (2008) Abeta peptide interactions with isoflurane, propofol, thiopental and combined thiopental with halothane: a NMR study. Biochim Biophys Acta 1778: 2633–2639. 18639516 10.1016/j.bbamem.2008.07.002

[27] Mandal PK, Fodale V (2009) Isoflurane and desflurane at clinically relevant concentrations induce amyloid beta-peptide oligomerization: an NMR study. Biochem Biophys Res Commun 379: 716–720. 19116131 10.1016/j.bbrc.2008.12.092

[28] Planel E, Richter KE, Nolan CE, Finley JE, Liu L, et al (2007) Anesthesia leads to tau hyperphosphorylation through inhibition of phosphatase activity by hypothermia. J Neurosci 27: 3090–3097. 17376970 10.1523/JNEUROSCI.4854-06.2007 PMC6672474

[29] Planel E, Krishnamurthy P, Miyasaka T, Liu L, Herman M, et al (2008) Anesthesia-induced hyperphosphorylation detaches 3-repeat tau from microtubules without affecting their stability in vivo. J Neurosci 28: 12798–12807. 19036972 10.1523/JNEUROSCI.4101-08.2008 PMC2610528

[30] Planel E, Bretteville A, Liu L, Virag L, Du AL, et al (2009) Acceleration and persistence of neurofibrillary pathology in a mouse model of tauopathy following anesthesia. FASEB J 23: 2595–2604. 19279139 10.1096/fj.08-122424 PMC2717763

[31] Whittington RA, Papon MA, Chouinard F, Planel E (2010) Hypothermia and Alzheimer’s disease neuropathogenic pathways. Curr Alzheimer Res 7: 717–725. 20678067 10.2174/156720510793611646

[32] Run X, Liang Z, Gong CX (2010) Anesthetics and tau protein: animal model studies. J Alzheimers Dis 22 Suppl 349–55. 20858970 10.3233/JAD-2010-100813

[33] Papon MA, Whittington RA, El-Khoury NB, Planel E (2011) Alzheimer’s disease and anesthesia. Front Neurosci 4: 272. 21344011 10.3389/fnins.2010.00272 PMC3034231

[34] Whittington RA, Virag L, Marcouiller F, Papon MA, El Khoury NB, et al (2011) Propofol directly increases tau phosphorylation. PLoS One 6: e16648. 21304998 10.1371/journal.pone.0016648 PMC3031597

[35] Dong Y, Wu X, Xu Z, Zhang Y, Xie Z (2012) Anesthetic isoflurane increases phosphorylated tau levels mediated by caspase activation and Abeta generation. PLoS One 7: e39386. 22745746 10.1371/journal.pone.0039386 PMC3379981

[36] Zhang Y, McLaughlin R, Goodyer C, LeBlanc A (2002) Selective cytotoxicity of intracellular amyloid beta peptide1–42 through p53 and Bax in cultured primary human neurons. J Cell Biol 156: 519–529. 11815632 10.1083/jcb.200110119 PMC2173346

[37] Zhang Y, Hong Y, Bounhar Y, Blacker M, Roucou X, et al (2003) p75 neurotrophin receptor protects primary cultures of human neurons against extracellular amyloid beta peptide cytotoxicity. J Neurosci 23: 7385–7394. 12917374 10.1523/JNEUROSCI.23-19-07385.2003 PMC6740455

[38] Liang G, Wang Q, Li Y, Kang B, Eckenhoff MF, et al.. (2008) A presenilin-1 mutation renders neurons vulnerable to isoflurane toxicity. Anesth Analg 106: 492–500, table of contents. 10.1213/ane.0b013e3181605b71 18227305

[39] Wei H, Liang G, Yang H, Wang Q, Hawkins B, et al (2008) The common inhalational anesthetic isoflurane induces apoptosis via activation of inositol 1,4,5-trisphosphate receptors. Anesthesiology 108: 251–260. 18212570 10.1097/01.anes.0000299435.59242.0e

[40] Zhen Y, Dong Y, Wu X, Xu Z, Lu Y, et al (2009) Nitrous oxide plus isoflurane induces apoptosis and increases beta-amyloid protein levels. Anesthesiology 111: 741–752. 19741497 10.1097/ALN.0b013e3181b27fd4 PMC2797570

[41] Dong Y, Zhang G, Zhang B, Moir RD, Xia W, et al (2009) The common inhalational anesthetic sevoflurane induces apoptosis and increases beta-amyloid protein levels. Arch Neurol 66: 620–631. 19433662 10.1001/archneurol.2009.48 PMC2748878

[42] Olney JW, Young C, Wozniak DF, Ikonomidou C, Jevtovic-Todorovic V (2004) Anesthesia-induced developmental neuroapoptosis. Does it happen in humans? Anesthesiology 101: 273–275. 15277906 10.1097/00000542-200408000-00004

[43] Hudson AE, Hemmings HC Jr (2011) Are anaesthetics toxic to the brain? Br J Anaesth 107: 30–37. 21616941 10.1093/bja/aer122 PMC3159425

[44] Zhang Y, Dong Y, Wu X, Lu Y, Xu Z, et al (2010) The mitochondrial pathway of anesthetic isoflurane-induced apoptosis. J Biol Chem 285: 4025–4037. 20007710 10.1074/jbc.M109.065664 PMC2823544

[45] Mallick B, Ghosh Z (2011) A complex crosstalk between polymorphic microRNA target sites and AD prognosis. RNA Biol 8: 665–673. 21659796 10.4161/rna.8.4.15584

[46] Fodale V, Santamaria LB (2003) Drugs of anesthesia, central nicotinic receptors and post-operative cognitive dysfunction. Acta Anaesthesiol Scand 47: 1180; author reply 1181. 10.1034/j.1399-6576.2003.00226.x 12969118

[47] Su D, Zhao Y, Wang B, Xu H, Li W, et al (2011) Isoflurane-induced spatial memory impairment in mice is prevented by the acetylcholinesterase inhibitor donepezil. PLoS One 6: e27632. 22114680 10.1371/journal.pone.0027632 PMC3219671

[48] Rodriguez JA, Buzaleh AM, Fossati M, Azcurra J, Batlle AM (2002) The effects of some porphyrinogenic drugs on the brain cholinergic system. Cell Mol Biol (Noisy-le-grand) 48: 103–110. 11929041

[49] Fodale V, Quattrone D, Trecroci C, Caminiti V, Santamaria LB (2006) Alzheimer’s disease and anaesthesia: implications for the central cholinergic system. Br J Anaesth 97: 445–452. 16950812 10.1093/bja/ael233

[50] Perry E, Walker M, Grace J, Perry R (1999) Acetylcholine in mind: a neurotransmitter correlate of consciousness? Trends Neurosci 22: 273–280. 10354606 10.1016/s0166-2236(98)01361-7

[51] Zhang Y, Goodyer C, LeBlanc A (2000) Selective and protracted apoptosis in human primary neurons microinjected with active caspase-3, -6, -7, and -8. J Neurosci 20: 8384–8389. 11069945 10.1523/JNEUROSCI.20-22-08384.2000 PMC6773170

[52] McKhann G, Drachman D, Folstein M, Katzman R, Price D, et al (1984) Clinical diagnosis of Alzheimer’s disease: report of the NINCDS-ADRDA Work Group under the auspices of Department of Health and Human Services Task Force on Alzheimer’s Disease. Neurology 34: 939–944. 6610841 10.1212/wnl.34.7.939

[53] Hyman BT, Trojanowski JQ (1997) Consensus recommendations for the postmortem diagnosis of Alzheimer disease from the National Institute on Aging and the Reagan Institute Working Group on diagnostic criteria for the neuropathological assessment of Alzheimer disease. J Neuropathol Exp Neurol 56: 1095–1097. 9329452 10.1097/00005072-199710000-00002

